# Exploring Responses to Art in Adolescence: A Behavioral and Eye-Tracking Study

**DOI:** 10.1371/journal.pone.0102888

**Published:** 2014-07-21

**Authors:** Federica Savazzi, Davide Massaro, Cinzia Di Dio, Vittorio Gallese, Gabriella Gilli, Antonella Marchetti

**Affiliations:** 1 Research Unit on Psychology of the Art, Department of Psychology, Università Cattolica del Sacro Cuore, Milan, Italy; 2 Research Unit on Theory of Mind, Department of Psychology, Università Cattolica del Sacro Cuore, Milan, Italy; 3 Department of Neuroscience, University of Parma, Parma, Italy; 4 Department of Art History and Archaeology, Columbia University, New York, New York, United States of America; 5 IIT (Italian Institute of Technology) Brain Center for Social and Motor Cognition, Parma, Italy; Centre de Neuroscience Cognitive, France

## Abstract

Adolescence is a peculiar age mainly characterized by physical and psychological changes that may affect the perception of one's own and others' body. This perceptual peculiarity may influence the way in which bottom-up and top-down processes interact and, consequently, the perception and evaluation of art. This study is aimed at investigating, by means of the eye-tracking technique, the visual explorative behavior of adolescents while looking at paintings. Sixteen color paintings, categorized as dynamic and static, were presented to twenty adolescents; half of the images represented natural environments and half human individuals; all stimuli were displayed under aesthetic and movement judgment tasks. Participants' ratings revealed that, generally, nature images are explicitly evaluated as more appealing than human images. Eye movement data, on the other hand, showed that the human body exerts a strong power in orienting and attracting visual attention and that, in adolescence, it plays a fundamental role during aesthetic experience. In particular, adolescents seem to approach human-content images by giving priority to elements calling forth movement and action, supporting the embodiment theory of aesthetic perception.

## Introduction

In 2010 “Studio 13/16” was opened in the Centre Pompidou (Paris, Piano and Rogers, 1977), one of the most important contemporary art museums and cultural centers in Europe. “Studio 13/16” is a space specifically designed for teenagers that offers workshops and ateliers in all fields of contemporary creativity (plastic arts, design, graphics, music, performance, dance, digital art, and street art). This interesting initiative is based on the contemporary idea that adolescents are to be conceived as a separate category of art viewers, with specific interests, tastes and needs.

To our knowledge, in the fields of art and psychology the study of the way in which adolescents perceive and evaluate art has been almost neglected or limited to the study of the effects of art therapies on clinical cases [1]–[4]. Developmental psychology has mostly focused on the way young children reason about art [5], greatly disregarding the development of art perception occurring at an older age. Considering the numerous and important changes that characterize adolescence at physical, psychological and social levels, it is likely that interesting turning points may occur in art perception during the developmental passage from childhood to adulthood. For example, during this period there are changes in visual perception that are mostly affected by biological, emotional and cognitive transformations typical of this age (for reviews on changes in brain structures and their consequences on behavior in adolescence see [6], [7]). It is thus plausible to hypothesize that adolescents may experience conventional pictorial art in a peculiar way. This hypothesis, on which the present research is based, revolves around one core psychological assumption: the physical and psychological changes adolescents undergo are prominent in influencing the perception of art and may well be accompanied by the maturation of notions and rules upon which judgments, including aesthetic judgments, are formulated.

The processes governing aesthetic experience in visual arts have been investigated through the analysis of visual behavior, such as the analysis of eye-movements (see [8]–[11]). Within these studies, understanding the interchange between “bottom-up” and “top-down” processes has played a central role [11]–[15]. According to the classic definition of these processes, bottom-up processes are usually mediated by the psychophysical (i.e., color and graphic traits expressing motion, such as curves, edges, lines, contrast) and organizational (i.e., symmetry, balance and complexity) properties of a stimulus [16], [17]. Instead, top-down processes are classically associated with factors, such as one's cultural background and education, the cognitive task under which the artworks are viewed and one's degree of expertise in the arts [18]. In other words, bottom-up processes are generally induced by low-level visual features that play a crucial role in guiding visual behavior during aesthetic experience (see, for example, [19]–[23]). Instead, top-down processes, such as contextual, social and cultural aspects of an image, are classically referred to as elements in a visual stimulus operating in a top-down fashion to elicit the interest of the viewer towards an artistic artifact (see, for example, [14], [24], [25]). Within these factors, also the semantic content of a visual artifact may mediate top-down processes driving the viewer's attention on specific areas of interest during aesthetic experience [10].

The few eye-tracking studies that consider the role of bottom-up and top-down processes on children and adolescents' perception of scenes and faces show that young people are susceptible to the influence of bottom-up more than that of top-down processes (for reviews, see [26], [27]). As described in Kramer et al. [28], only starting at 8 years of age, children begin exercising the ability to exert top-down control in opposition to stimulus-driven bottom-up influence on attentional capture. Furthermore, the ability to maintain multiple top-down sets like, for example, to inhibit eye movements to salient stimuli moving the eyes in the opposite direction, appears to take even longer to develop. Also brain-imaging research supports the aforementioned results. In Luna and colleagues' [29] experiment, 8 to 30 years olds' ability to voluntarily suppress context-inappropriate behavior was investigated with functional brain imaging while subjects performed oculomotor suppression. The results showed that brain activation in several cortical and subcortical regions increased progressively from childhood to adulthood. Adolescents further showed a great prefrontal activation in anti-saccade performance, suggesting that top-down modulation of reflexive/impulsive responses is not fully efficient - although already present - in adolescence.

Altogether, the findings of these studies suggest that – within the classical perspective on the interaction of bottom-up and top-down processes – adolescence is a critical developmental stage during which top-down processes begin to emerge affecting perception, although their influence is still not fully developed. As a matter of fact, also anatomical neuroimaging studies showed that the prefrontal areas of the brain are among the last to mature [30]–[32]. In other words, it is as if adolescents need to integrate all their psychological and physical changes into a coherent body image through a complex process of definition into a new adult identity. Not by chance, during adolescence many psychological body-related disorders usually arise, such as eating disorders [33], affective and anxiety disorders [34], substance abuse [35], self-harm [36], and risk-taking [37].

The peculiar interaction between top-down and bottom-up processes may appear not only in the redefinition of the representation of one's own body, but also in the perception of the body represented in images and photos. The brain-imaging work by Monk and colleagues [38] showed that, when looking at details of faces, adolescents are affected by emotionally evocative cues. More specifically, adolescents show a high frontal activity – typically involved in attentional tasks when asked to pay attention to non-emotional aspects of the face, such as the nose of fearful faces. This activation pattern suggests that, at this age, neglecting the emotional aspects of a stimulus requires a high attentional effort [6]. These results bring about the idea that adolescents' distinctive perception of salient visual elements in a figure, possibly affected by a specific body schema, may also influence the way in which artworks are explored and eventually evaluated.

The maturation of the relationship of top-down and bottom-up influence on how the stimuli are processed and perceived may reasonably also extend to art processing and evaluation. In particular, this maturation may affect explicit aesthetic judgment of artwork. Aesthetic experience for (visual) artworks possibly starts from a visual analysis of the stimulus and then undergoes different processing stages of which explicit aesthetic judgment is considered as the output of cognitive processing and aesthetic appraisal [18], [39], [40]. In this respect, it has been hypothesized [41]–[43] that people acquire a set of concepts, beliefs and desires on visual arts that are used to develop reasoning and to formulate judgments on the recognition and on the beauty of artworks. Thus, aesthetic evaluation develops within a process of acquisition characterized by progressive normative stages according to the chronological age. Aesthetic judgments in young children are mainly based on content and personal beliefs, and, later, also on references to beauty and realism in representation. Growing up, children become able to aesthetically judge an artwork by focusing on the understanding of the artists' feelings and thoughts while producing that artwork; successively their judgment focuses more and more on the artistic style and form as well as on the underlying concepts. This interpretative activity, which implies an intentional stance towards the art, is a lifelong endeavor.

Interestingly, the peculiar interplay of bottom-up and top-down processes in the way young people approach art comes also from recent evidence in the field of museum education. New educational perspectives oppose to previous models for communication that emphasized the transfer of information to passive receivers using a didactic approach. The more recent constructivist approaches [44], [45], instead, acknowledge the importance of personal agency and active learning [46], [47]. These approaches are meant to let young visitors directly experience the features of artworks and personally discover the meaning in paintings [48]. Young visitors are in fact involved in educational practices empowering critical thinking and enhancing a co-construction of meanings.

A factor that may intervene in the specific interaction between bottom-up and top-down processes is the mechanism of embodiment. According to this idea, the peculiar perception of one's own body during adolescence may play an important role in way adolescents perceive and evaluate art. In a recent study [15] the visual exploration patterns of adult participants during art observation and evaluation was interpreted as guided by bodily mechanisms influenced by specific top-down processes. The relationship between top-down and bottom-up processes seemed to stem from the salience of the content represented in the painting. In fact when a human being (and not a nature content) was portrayed, content-related processes prevailed over low-level visually-driven bottom-up processes in guiding the observers' explorative pattern. This effect was interpreted in terms of embodied simulation (see also [49]).

Using the eye-tracking technique, in the present study we focused on how bottom-up and top-down processes interact while a group of adolescents visually explored and aesthetically appraised paintings. We considered visual behavior as an index of overt selection expressing the link between the area observed with the viewer's interest [50]. Stimuli were presented for a duration of 3 s, shown to be a reliable period to form and express a stable evaluation of the artwork in previous works [51]–[54]. It was shown that the perception of pictorial properties, such as symmetry and balance, can be detected after only 50 ms glance at the visual stimulus. Locher and colleagues [55] found that, about 2 s after the onset of the pictorial stimuli, viewers were able to provide a holistic description of the characteristics of the artworks in response to their expressive qualities, style and form. Additionally, the authors provided evidence that the pleasantness ratings of the paintings obtained following a brief glance correlated with exposition to the artworks for unlimited viewing (see also the preliminary study for stimulus selection in Di Dio et al. [56]).

Our main hypothesis is that adolescents pass through changes due to physical maturation which may intervene on the way bottom-up and top-down processes interact also in the perception and evaluation of art. We presented adolescents with color paintings representing natural environments and human subjects (Content), categorized as dynamic and static (Dynamism) on the basis of the presence of visual features engendering movement perception (such as orientation, curvature and convergence of lines, see [57]). All the stimuli were displayed under aesthetic and movement judgment tasks (Task). Our results generally showed that when experiencing a pictorial artwork adolescents are attracted by elements that most probably evoke a bodily simulation in the beholder.

### Experimental aims

We were interested in exploring the way in which adolescents' aesthetic experience would be affected by the content and dynamism represented in visual artworks and by two different judgments tasks. Furthermore, we were interested in verifying a possible relationship between visual exploration during aesthetic experience and the explicit judgment adolescents later express. Our main hypothesis was that adolescents' judgment and visual exploration may be affected by physical maturation processes. We expected judgment and visual exploration to reflect adolescents' interest for body and dynamism associated to the action. Therefore, as for the judgments expressed by adolescents, we expect a recognition of the degree of movement represented in paintings and higher aesthetic ratings addressed to dynamic images with respect to static ones. Secondly, we hypothesize that adolescents' visual pattern would be much influenced by dynamic cues and focused on few salient areas on human content images (human body) with respect to nature content images.

## Methods

### Participants

Twenty Italian adolescents (12 females, 8 males; mean age  = 13 years; range  = 12–15) took part in this study. All the participants had normal or corrected to normal visual acuity. Participating families were recruited at a secondary school in Milan. After being informed about the purpose and procedure of the study, parents could contact our research team to agree upon their child's participation in the study. Adolescents were tested at the Department of Psychology, Università Cattolica del Sacro Cuore in Milan. The parents were rewarded for their child's participation with a 40 Euros shopping voucher.

### Ethics Statement

The participants' parents gave their written informed consent to the experimental procedure. The study was approved by the Local Ethic Committee (Università Cattolica del Sacro Cuore, Milan).

### Visual Stimuli, Procedure and Tasks

Sixteen digital images of paintings were randomly chosen, within each one of the four categories from the database of a previous work [15] in which, researchers – starting from a set of 100 paintings – selected the 40 less known artworks. The original stimuli were categorized in static and dynamic on the basis of the represented movement as rated by independent judges. Additionally, half of the stimuli represented human figures and the other half landscapes. According to this categorization, the following groups of images were used for this study: 4 dynamic human images, 4 static human images, 4 dynamic nature images, and 4 static nature images (for the details of the paintings used in this study see Table S2 in Supplementary Information). The aspect ratio of the paintings was preserved. Image sizes ranged from 495×812 to 788×524 pixels. The visual angle covered by the images measured on average 20° – both on horizontal and vertical axes – so that stimuli were presented within the 30° of focal visual field and participants could freely move their eyes without turning their head. Participants looked at the presentation of the stimuli created with Tobii Studio 1.3 software (Tobii Technology AB) on a computer monitor at a distance of 70 cm. The presentation of the stimuli was repeated twice: under aesthetic judgment (AJ) and under movement judgment (MJ) tasks. The two tasks were presented in separate sessions and were counterbalanced across participants. Each image was introduced by a 1-second central cue (black cross on white screen) and was shown for 3 seconds, a suitable period of exposition to an artwork in order to produce a reliable aesthetic judgment [51]–[56]. Stimuli were presented in a random order. Participants' eye-movements were recorded through Tobii X120 Eye-Tracker (see next section). A calibration session was always presented to participants before each task.

At the end of each trial participants were shown a task-related question (Aesthetic Judgment task “How beautiful is the painting you just saw?”; Movement Judgment task “In your opinion, to what degree the painting you just saw expresses movement?”) to which they gave an oral answer using a seven-point Likert scale. The experimenter manually recorded the answers. As an answer was recorded, the experimenter started the new trial. Each eye-tracking session lasted approximately 5 minutes.

### Eye-Tracking apparatus

Tobii Eye-Tracker X120 set was used in order to record data on eye-movements. The software (Tobii Studio 1.3) processed eye-movements in terms of number and duration of fixations (when a point of the external world is located on the fovea for approximately 300 ms) and observations (each time a cluster is entered and exited, see below). The software progressively created clusters with high density of fixations by means of the robust clustering algorithm [58]. Clusters were created by aggregating the fixation patterns of each participant across the same image. A final recapitulatory image of every stimulus was then created by the software superimposing a graphic representation of the areas with high concentrations of fixation points for the total number of participants. For each stimulus, two aggregations of fixation points across participants were made: one for the stimulus observed under the aesthetic judgment task and the other for the same stimulus observed under the movement judgment task. Hence, each of the 20 participants could approximately make 10 fixations in the 3-seconds period of observation on each image, resulting in a total of almost 200 fixations per image across participants.

Data were normalized in relation to the area of images and of the size of each cluster. Eye-movements data were extracted starting from 0.2 ms in order to control possible bias produced by the central cue preceding each image.

### Analysis

Between-effects were explored by the mean of a univariate GLM. As for the analysis of within- and between-effects fixed-effects, an ANOVA model was used. This model was chosen because robust and able to provide very reliable results even with small sample sizes [59]–[61]. For the multiple comparisons, the Sidak correction was applied.

#### Global pattern analysis

Following Massaro and colleagues' [15] method, analyses of eye-tracking data (total number of fixations per image and mean duration of a fixation) were firstly carried out within the total number of clusters formed in the paintings (see Tab. S1 in the Supporting information for a detailed description of the variables).

#### Cluster analysis

Gazing behavior within each cluster was analyzed considering the minimum number of four clusters (Regions of Interest, ROI) built across all images (range 4–11).

## Results and Discussion

### Behavioral analysis

**Figure 1:**
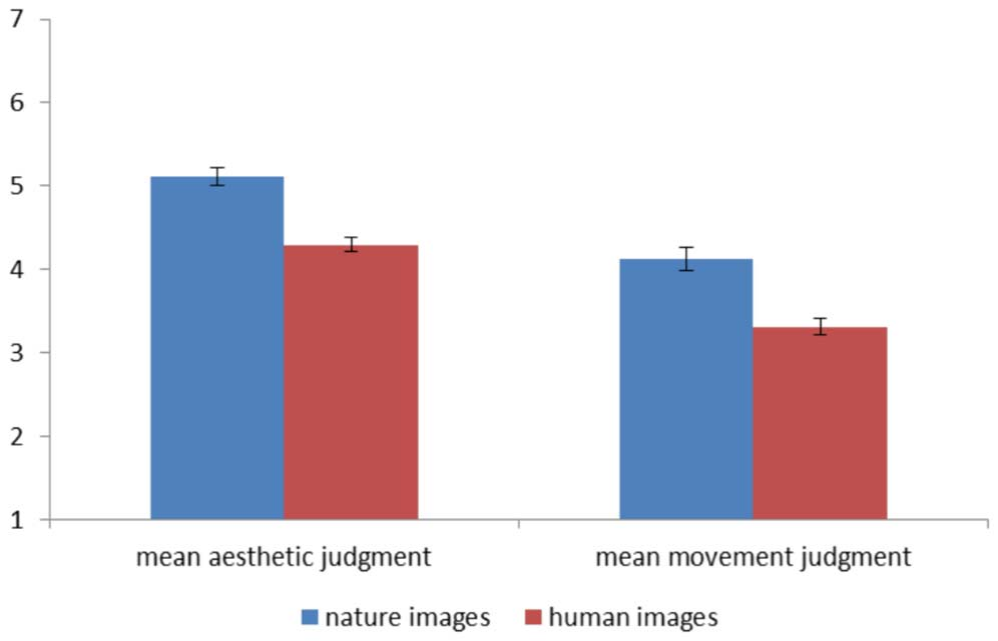
Aesthetic and Movement ratings for paintings representing nature and human figures.

Within the tasks of aesthetic judgment (AJ) and movement judgment (MJ), a 2×2 General Linear Model (GLM) analysis on the behavioral ratings, with 2 levels of stimulus Content (human [H] vs. nature [N]) and 2 levels of stimulus Dynamism (dynamic [D] vs. static [S]), was carried out. No significant results are not reported. As far as AJ task is concerned, the results revealed a main effect of Content (F_(1 19)_ = 14.214; p<.01, η^2^ = .43, δ = .95; N>H). More specifically, images representing landscapes were preferred over images portraying human figures (Fig. 1 and Table 1).

**Table 1:** Mean behavioral ratings (and standard error – SE) per sub-category for AJ and MJ.

		Top-Down					
		Mean (SE)					
Judgment (1–7 Likert scale)		Movement (MJ)			Aesthetic (AJ)		
		Nature	Human		Nature	Human	
**Bottom-up**	**Static**	3.01(.35)	2.41(.28)	***2.71(.29)***	4.99(.22)	4.26(.20)	***4.62(1.55)***
Mean (SE)	**Dynamic**	5.25(.26)	4.21(.20)	***4.73(.20)***	5.24(.24)	4.32(.16)	***4.78 (.18)***
		***4.13(.28)***	***3.31(.19)***		***5.11(.21)***	***4.29(.17)***	

A tentative explanation for higher aesthetic evaluation ascribed to nature images compared to human figure images may account for the idea that the representation of landscapes is less influenced by historical changes than the representation of humane figures. In fact, nature may have a prototypical appearance, whereas the human body –even though it elicits a bodily empathetic experience in the beholder– may be not recognized as responding to contemporary canons, particularly to the eyes of young viewers. This top-down influence may decrease the aesthetic value of images representing humans.

With reference to MJ task, results showed a main effect of Dynamism (F_(1 19)_ = 82.832; p<.001, η^2^ = .81, δ = 1.0; D>S) and Content (F_(1 19)_ = 25.515; p<.001, η^2^ = .57, δ = 1.0; N>H). Coherently with the original stimuli categorization, dynamic images obtained higher movement ratings than static images. Additionally, paintings representing nature were judged on average as expressing more movement than those representing human figures (Fig. 1 and Table 1).

These results suggest that adolescents are sensible to low-level features in evaluating movement. The nature content may not attract participants' attention with the same strength of human images. According to Massaro and colleagues [15], bodily driven mechanisms would mainly affect the exploration of human images, supporting a more precise and modulated perception of movement. The perception of nature images would be mostly influenced by visual characteristics of the paintings possibly driven by low-level visual features. This idea would account for a higher recognition of movement in nature than human paintings.

### Eye-tracking global pattern analysis

#### Number of clusters

We carried out a univariate GLM analysis on the number of eye-fixation clusters (dependent variable) with Content (human [H] vs. nature [N]), Dynamism (dynamic [D] vs. static [S]), Judgment task (aesthetic judgment [AJ] vs. movement judgment [MJ]) as within-subject independent variables. No significant results are not reported. The results revealed a main effect of Content (F_(1 24)_ = 5.042; p<.05, η^2^ = .17, δ = .58; N>H), i.e. the number of clusters was lower in human (M = 5.69, SE = .43) than in nature (M = 7.06, SE = .43) images. No interaction effects were observed between any of the variables. These data suggest that the human content attracts gaze on few specific and meaningful areas as compared to nature content, independently of dynamism and task. Results are summarized in Table 2.

**Table 2:** Mean number of clusters (and standard error – SE) per sub-category.

		Top-Down					
		Mean (SE)					
		Movement (MJ)			Aesthetic (AJ)		
		Nature	Human		Nature	Human	
**Bottom-up**	**Static**	6.75(.87)	5.25(.87)	***6.00(.61)***	7.00(.87)	5.50(.87)	***6.25(.61)***
Mean (SE)	**Dynamic**	6.75(.87)	5.50(.87)	***6.13(.61)***	7.75(.87)	6.50(.87)	***7.13(.61)***
		***6.75(.61)***	***5.38(.61)***		***7.37(.61)***	*6.00(.61)*	

#### Total number of fixations and fixation mean duration

Total number of fixations and mean duration of a fixation were explored with a 2×2×2 GLM with Content (human [H] vs. nature [N]), Dynamism (dynamic [D] vs. static [S]), and Judgment task (aesthetic judgment [AJ] *vs*. movement judgment [MJ]) as independent within variables.

Considering the mean duration of a single-eye-fixation per image, significant main effects were found for Content (F_(1 19)_ = 9.069; p<.01, η^2^ = .32, δ = .81; H>N) and Dynamism (F_(1 19)_ = 14.445; p<.01, η^2^ = .43, δ = .95; S>D). Specifically, eye-fixation was on average longer on human images (M = .41, SE = .03) than on nature images (M = .35, SE = .02) and on static images (M = .41, SE = .02) than on dynamic images (M = .35, SE = .02). An interaction between Content and Dynamism (F_(1 19)_ = 4.851; p<.05, η^2^ = .20, δ = .55) was also found. Human static images (M = .45, SE = .03) required longer mean fixations than nature static images (M = .36, SE = .02; F_(1 19)_ = 10.382; p<.01, η^2^ = .35, δ = .86; HS>NS) and human static images (M = .45, SE = .03) were observed with longer mean fixations than human dynamic images (M = .37, SE = .37; F_(1 19)_ = 14.189; p<.01, η^2^ = .43, δ = .95; HS>HD). As for the total number of eye-fixations no significant results were found. Results are summarized in Tables 3 and 4.

**Table 3:** Mean number of fixations (and standard error – SE) on the total clustered area of images per sub-category.

		Top-Down					
		Mean (SE)					
		Movement (MJ)			Aesthetic (AJ)		
		Nature	Human		Nature	Human	
**Bottom-up**	**Static**	3.32(.29)	3.53(.35)	***3.42(.24)***	2.86(.25)	2.96(.16)	***2.91(.17)***
Mean (SE)	**Dynamic**	2.69 (.19)	3.07(.26)	***2.88(.17)***	3.70(.50)	3.26(.27)	***3.48(.33)***
		***3.01(.17)***	***3.30(.24)***		***3.28(.30)***	***3.11(.18)***	

**Table 4:** Mean fixations duration (and standard error – SE) (in seconds) on the total clustered area of the images per sub-category.

		Top-Down					
		Mean (SE)					
		Movement (MJ)			Aesthetic (AJ)		
		Nature	Human		Nature	Human	
**Bottom-up**	**Static**	.37(.03)	.44(.04)	**.** ***40(.03)***	.36(.03)	.46(.04)	**.** ***41(.03)***
Mean (SE)	**Dynamic**	.35(.02)	.39(.03)	**.** ***37(.02)***	.32(.02)	.35(.02)	**.** ***33(.02)***
		**.** ***36(.03)***	**.** ***42(.03)***		**.** ***34(.02)***	**.** ***41(.03)***	

These results are in line with those on the number of clusters showing lower number of clusters on human than on nature images. In effect, longer fixations on human images than on nature images suggest that the formers were likely to contain more meaningful elements than nature images. Furthermore, within the human images, the absence of dynamic cues probably induced a longer exploration of those meaningful elements.

### Eye-tracking cluster analysis

Eye tracking variables were explored within each ROI using 2×2×2 GLM models with Content (human [H] vs. nature [N]), Dynamism (dynamic [D] *vs*. static [S]), and Judgment task (aesthetic judgment [AJ] *vs*. movement judgment [MJ]) as independent within variables. No significant results are not reported.

#### Cluster size

The results showed a main effect of Content (F_(4 21)_ = 4.476; p<.01, η^2^ = .46, δ = .87; N>H). Namely, the extension of ROI 2 (F_(1 24)_ = 6.022; p<.05, η^2^ = .20, δ = .65) and ROI 4 (F_(4 21)_ = 5.477; p<.05, η^2^ = .19, δ = .61) was significantly greater in nature than in human images. No effects were found within ROIs 1 and 3. See Table 5 for results. The finding of narrower clusters –together with longer fixations and less clusters– in human than in nature images clearly shows how the human body evokes fixations on specific and meaningful areas.

**Table 5:** Clusters size (%) in image representing human *vs*. nature content.

	Human				Nature			
	Minimum	Maximum	*Mean*	*SD*	Minimum	Maximum	*Mean*	*SD*
**Cluster 1**	.41	4.89	***2.03***	*1.09*	.21	4.47	***2.37***	*1.07*
**Cluster 2**	.09	3.32	***1.64***	.*82*	.87	4.76	***2.63***	*1.25*
**Cluster 3**	.34	3.99	***1.28***	.*94*	.22	3.92	***1.79***	*1.19*
**Cluster 4**	.04	1.89	**.** ***83***	.*64*	.09	4.23	***1.60***	*1.25*

#### Number and duration of fixations and observations

Time to first fixation, fixation number and duration, observation number and duration were considered within each of the 4 first ROIs (see Table S1 in the supplementary material for a detailed description of the variables and Table 6 for statistics).

**Table 6:** GLM main effects for fixations and observations of the first 4 ROI's.

Indexes	ROI	Content						Dynamism						Judgment Task					
			F	df	*p*	η^2^	δ		F	df	*p*	η^2^	δ		F	df	*p*	η^2^	δ
**Fixations number**	1	-						-						MJ>AJ	7.211	1,19	.015	.27	.72
	2	H>N	34.269	1,19	.000	.64	1.0	S>D	27.327	1,19	.000	.59	.99	MJ>AJ	13.043	1,19	.002	.40	.93
	3	-						-						-					
	4	-						-						-					
**Observations number**	1	-						-						MJAJ	5.931	1,19	.025	.24	.64
	2	H>N	40.968	1,19	.000	.68	1.0	S>D	11.261	1,19	.003	.37	.89	MJ>AJ	11.936	1,19	.003	.39	.91
	3	-						-						-					
	4	-						-						-					
**Fixations duration**	1	H>N	13.893	1,19	.001	.42	.94	-						MJ>AJ	5.454	1,19	.031	.22	.60
	2	H>N	91.000	1,19	.000	.83	1.0	S>D	63.246	1,19	.000	.77	1.0	MJ>AJ	15.813	1,19	.001	.45	.96
	3	H>N	4.767	1,19	.042	.20	.54	-						-					
	4	-						-						-					
**Observations duration**	1	H>N	13.895	1,19	.001	.42	.94	-						-					
	2	H>N	113.808	1,19	.000	.86	1.0	S>D	71.467	1,19	.000	.79	1.0	MJ>AJ	14.107	1,19	.001	.43	.94
	3	-						-						-					
	4	-						-						-					
**First Fixation Duration**	1	-						S>D	14.163	1,19	.001	.43	.95	-					
	2	H>N	105.582	1,19	.000	.85	1.0	S>D	45.499	1,19	.000	.70	1.0	MJ>AJ	8.868	1,19	.008	.32	.81
	3	H>N	6.085	1,19	.023	.24	.65	-						-					
	4	-						-						-					
**Time to first fixation**	1	H>N	10.821	1,19	.004	.39	.87	-						-					
	2	-						-						-					
	3	-						-						-					
	4	-						-						-					

In terms of duration of fixations and observations, in ROIs 1, 2 and 3 results showed a main effect of stimulus Content (H>N): in ROIs 1 and 2, fixations and observations duration were longer for human than for nature images. In ROI 3 this effect was present only for fixations. Also the duration of the first fixation in ROIs 2 and 3 was longer on human than on nature images. In particular, the number of fixations and observations was higher on human images than on nature images in ROI 2.

As far as the time-to-first-fixation is concerned, a main effect of Content (H>N) was also found in ROI 1: the time necessary to enter into the first ROI was longer in human-content than in nature-content stimuli. The human body seems to exert a stronger fixation-evoking power in the first clustered areas than nature. Moreover, the representation of human body seems to activate a prototypical representation that causes a longer search for few specific body elements than in nature images.

Additionally, a main effect of Dynamism was found in ROI 2 (S>D): the number and duration of fixations and observations were higher in static images than in dynamic images. Finally, a main effect of Judgment task (MJ>AJ) was also found in ROIs 1 and 2. In particular, during MJ task the number and duration of fixations and observations were higher than during AJ task (except for the duration of observations and of the first fixation: for these indexes this effect was present only in ROI 2). This result may suggest two complementary explanations: adolescents may be massively sensitive to movement information and, consequently, they may need a strong attentional effort in order to express a modulated and precise movement evaluation. Moreover adolescents may show a proclivity to judge several aspects of their life in terms of pleasantness in an unmediated and pre-reflective way.

### Content Analysis

Considering only human-content paintings, the content of each ROI was analyzed. In particular, the ROIs were categorized on the basis of the specific portion of the body bounded by the ROI itself (face, limbs, trunk or mixed content – face+limbs or face+trunk –, not on human body).

**Figure 2:**
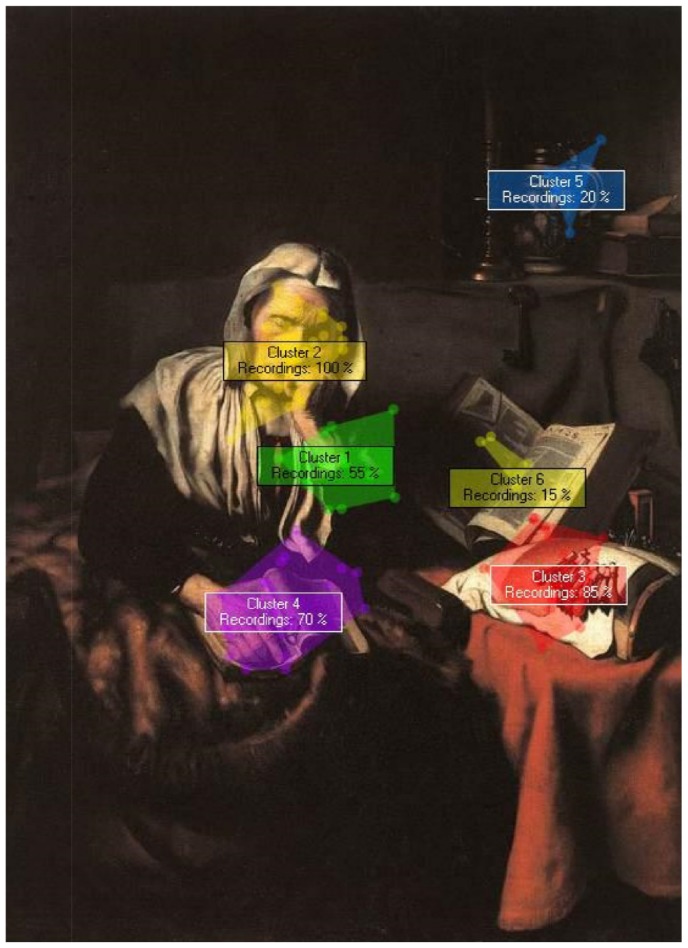
Example of cluster distributions on a human static image (Old Woman Dozing, Nicolaes Maes, 1656). Cluster number represents the temporal order in which clusters have been shaped considering the pattern of fixations from all participants. The percentage of participants looking in each cluster is reported.

The results showed that in ROI 1 limbs were the most viewed area (37.5%) followed by trunk (31.3%), face (12.5% face +12.5% mixed content  = 25%) and out-of-the-body (6.3%); in ROI 2, the face was the predominant explored area (43.8% face +31.3% mixed content  = 75%) followed by the limbs (12.5%) and out-of-the-body (12.5%). On the whole, the face area was the first clustered area (ROI 1) in 25% of the cases; this value rose to 95% if also considering the content of ROI 2 (Fig. 2).

Additionally, the results revealed that, in ROIs 3 and 4, the face was never explored. Instead, in ROI 3 adolescents looked out of the human body in 43.8% of the cases, followed by limbs (37.5%) and by trunk (18.8%). In ROI 4 attention was mostly drawn by limbs (62.5%) and by elements out-of-the-body (37.5%). Content analysis shows adolescents' peculiar way of exploring the human body. In fact, adolescents appear to be firstly attracted by body parts, such as limbs, and only later by the face. In this light, it is possible to hypothesize that adolescents are firstly interested on the portrayed action and only later on the emotions expressed by the subject's face, thus entering in relation with the human content in a very physical way.

## General Discussion

In the present study we explored, by means of psychophysical measures, adolescents' response to visual art. The modern trend is of considering adolescents as a separate group of art viewers that experience art in a distinctive manner. This assumption is based on the hypothesis that adolescence is characterized by changes in the body that may also affect psychological processes and, more specifically, the interaction between bottom-up and top-down processes in the production of a coherent aesthetic experience. Using the eye-tracking technique, we therefore investigated the interplay between bottom-up and top-down processes when adolescents visually explored and explicitly assessed representational paintings. Paintings were categorized as a function of variables affecting both top-down and bottom-up processes and, namely, by their content (landscapes or human beings - Content) (top-down-processes) and by expressed movement (static or dynamic - Dynamism) (bottom-up processes). Participants' responses to the painting were recorded in two tasks: aesthetic and movement judgments.

With respect to the participants' explicit judgments to the paintings, results showed that adolescents judged nature paintings as more beautiful than paintings representing the human body. A possible explanation for the preference given to nature than to human content is that these two categories have a different historical connotation. In fact, the representation of landscapes is less influenced by the changing of times and may be easily recognized by contemporary people because they are close to the way nature environments are still. Instead, the representation of human figures is highly affected by time and fashion. This historical connotation in human figure representations may have decreased adolescents' aesthetic evaluation of images representing humans. The preference for nature stimuli could also be explained considering the role of low-level visual features in perception when observing nature images. In Massaro et al. [15], it was suggested that there may be differential effects of low-level visually-driven bottom-up processes on gazing behaviour as a function of painting content. In particular, when nature is represented, bottom-up processes appear to mostly affect gazing behaviour. On the other hand, when the represented content includes a human subject, bodily-driven content-related top-down processes prevail over low-level visually-driven bottom-up processes in guiding the observers' explorative pattern. It is possible to hypothesize that, in adolescents, the guidance of low-level visual features may also affect aesthetic judgment.

Adolescents also revealed a good discrimination of dynamic cues by differentiating between static and dynamic images in the judgment of movement, as posited in our first hypothesis.

Developmental models of aesthetic judgment, previously described [41]–[43], suggest that there is an evolution in the formulation of aesthetic judgment as a function of the progressive acquisition of concepts on visual arts. In consideration of these models, our results show that adolescents have already attained a set of beliefs on visual art that enable them to define what aesthetic beauty is and to evaluate it. On the basis of our findings, little can be said about the motivations guiding adolescents to express their aesthetic appraisals. However, it seems plausible to interpret our participants' aesthetic judgments as mainly based on their personal taste and not on a recursive reasoning that includes a complex set of components like: the artist's expressive abilities, intent and emotions, the artistic style, the cultural framework where the artwork is to be allocated and the observer's critical point of view [41], [42]. As a matter of fact, participants spent less time exploring paintings when asked to express an aesthetic than a movement judgment, showing a tendency to judge beauty on the basis of an unmediated and pre-reflective evaluation of pleasantness. Another cue suggesting that our group of adolescents did not fully reach the understanding of the concepts underlying the artwork is their preference (higher aesthetic evaluations) for nature compared to human-content paintings. Evaluation of nature contents requires less interpretative processing than artworks depicting human subjects that, on the contrary, require multifold levels of processing, that go beyond the mere pictorial depiction of the object.

Results from visual exploration indexes (number and size of clusters) generally showed that adolescents' visual behaviour was affected by content-related processes. In fact, by comparing the ways adolescents explored nature and human images, we found that visual exploration differed in the number and extension of the areas of interest as well as in the time spent inspecting those areas. While for human paintings visual behavior was concentrated on few and specific areas, for nature images participants explored a greater and more variable number of potential elements of attraction. This evidence suggests that, in adolescence, the human body exerts a strong power orienting and attracting visual attention. In fact, within eye-tracking studies, a higher concentration of fixations on specific parts of the painting indexes the attractive power of that part on eliciting beholders' attention [24]. Visual interest is an index of one's preference for a represented element. In our study, participants concentrated on restricted areas during visual exploration of human-content paintings, suggesting robust attractiveness on specific parts of the human body and confirming our second hypothesis. It is possible to interpret this result in light of the embodied simulation perspective in art perception [49]. According to this theory, the displaying of actions, sensations and emotions in artworks would activate basic mirror-like processes in the viewer: executing actions or experiencing emotions and sensations activate the same neural structures activated when we see others acting or expressing the same emotions and sensations [62], [63]. The human body represented in artworks seems to guide adolescents' attention in a specific and robust fashion.

As a matter of fact, the analysis of the eye-tracking variables representing initially observed areas not only confirmed that the human figure in the paintings strongly attracted attention (longer time spent on human than on nature content), but also that the content of some of these areas was highly meaningful (smaller clusters on human than on nature images): the human body firstly and strongly attracted the visual exploration of adolescents.

It addition, it is worth noting that adolescents' attention was firstly drawn on body parts usually involved in the execution of actions, such as limbs, and only later on the face, which is generally recognized as one of the most interesting and social relevant areas of the body [64], [65]. This evidence betrays a peculiar way of perceiving the body in adolescence. In fact, it seems that the body dimension is so relevant to adolescents that they are first interested in exploring what a person is doing (with their limbs) and, only later, what a person is thinking or feeling (through face expressions), independently of the task they are asked to perform. As previously described, visual exploration of paintings may be guided by embodied simulation of both actions and the expression of emotions. Adolescents visual behavior seems to be primarily guided by the effective actions that humans represented paintings are executing.

This evidence could be in line with the difficulties adolescents may present in processing at a conscious level personal bodily experiences, such as emotions [34]. In fact, they have to face a highly stressful period because of the physical, cognitive, and social changes they experience. Thus they risk to meet a delay in the development of the emerging skills of emotion regulation [66] and may present a low awareness of their own emotional state, as well as difficulties in putting in someone else's shoes and using an appropriate language to refer to the others' feelings. As a possible consequence of this way to enter in relation with others, they are firstly guided to explore actions and active interactions (limbs and trunk) than the expression of personal inner states (face).

This result is new also considering adults' way of exploring paintings representing human beings. Indeed, within a similar experiment [15], adults' attention was drawn firstly to face and only later to arms and legs. This discrepancy between the visual behavior of adults and adolescents in entering into relation with the human body gives support to the hypothesized role of pubertal development and the consequent peculiarity in body perception typical of adolescence. Adolescents seem to approach human images by giving priority to elements calling forth movement and action while adults are more driven by elements referring to feelings and thoughts.

Finally, adolescents' explorative visual behavior, as assessed in this study, may share some peculiarities with the way children with autism approach visual stimuli. Autism is a clinical condition characterized by impairments in social, representational and communication abilities, possibly related to a deficit in perceptual integration. It seems like some aspects of the typical development of visual processing may be heightened and exacerbated in autism. Recent evidence shows that children with autism present an altered visual processing characterized by detailed-oriented perception and reduced attentional zoom abilities (see, for example, [67]–[69]). These aspects of autistic children's visual behavior can be associated, to a certain extent, to the way in which adolescents of this study explored paintings representing human figures, focusing on restricted areas and being firstly attracted by body parts different than the face.

As far as movement judgment is concerned, participants made a greater effort (more and longer fixations and observations) to judge the sense of movement evoked by a painting than its aesthetics. It is likely that adolescents' explicit cognitive evaluation of movement required by the task suffered the massive sensitivity to movement and action. So, they probably had to make a strong effort in order to express a modulated and precise explicit movement evaluation.

Finally, our results bring about the idea that adolescents show some peculiarities when exploring and evaluating artistic images representing the human body, probably because of the relevance the body gains during this developmental phase. Firstly, considering results of the exploration of human-content images, adolescents seem to inhibit the guidance of the perception of movement even if they seem to be initially and primarily interested in the parts of the body involved in the execution of actions. They explored and re-explored static images more than dynamic images (mean duration single eye fixation on the total area of the images). This result may be read again in light of the low capability of adolescents to symbolically express what they ‘bodily’ experience. The greater interest for static images that seems to emerge from this result may be explained by adolescents' difficulty in translating at a reflexive level the movement of the body which they closely experience. Secondly, adolescents explicit evaluated nature content as more beautiful than human content images even though their visual behavior expressed a clear preference for the human body as previously described. In fact, on the one side, the answers to the two tasks revealed that nature images were preferred over human images. On the other side, eye-movements revealed the highly meaningfulness of human images and the immediate attractiveness of the body parts implied in actions. Adolescents firstly looked at what the depicted person is doing. Face begins to make sense only when the body does less. Finally, participants struggled more with movement judgments than with aesthetic evaluations.

Concluding, our data seem to support the art education perspective that emphasizes adolescents' discover of the meaning of art through global multisensorial involvement, instead of a didactic approach whereby intellectual information is given by skilled adults. By showing the close interplay between bottom-up and top-down processes when the object of the artistic representation is a human body, our data point out the importance of a bodily engagement with art by young people. Our results then suggest a possible role of relational and intersubjective processes on aesthetic experience in line with Freedberg and Gallese's theoretical hypothesis [49] according to which aesthetic experience is based on the activation of neural structures involved in social empathetic interaction. New educational approaches may then help adolescents experience art primarily through their body and develop a critical thinking about its meaning [47].

Our study may be a starting point for further research on aesthetic experience in adolescence. Future studies may use other content categories different from the ones used (landscapes and humans) in order to broaden the representativeness of stimuli. Furthermore, it could be interesting to present adolescents with representations of the human body selected within contemporary artworks to overcome the possible detached reaction towards historically connoted human representations as we hypothesized here.

## Supporting Information

Table S1 **Description of the variables used for the analysis of eye-movements and the relative ascribed interpretation (see Massaro et al., 2012).**
(DOC)

Table S2 **List of Paintings.**
(DOCX)
