# Supplementary material for: Exploring Responses to Art in Adolescence: A Behavioral and Eye-Tracking Study
Source: PLoS One. 2014 Jul 21;9(7):e102888. doi: 10.1371/journal.pone.0102888 (PMC4105571; doi:10.1371/journal.pone.0102888)
Supplement: Table S1 — Description of the variables used for the analysis of eye-movements and the relative ascribed interpretation (see Massaro et al., 2012). (DOC) [file pone.0102888.s001.doc]

**Table S1. Description of the variables used for the analysis of eye-movements and the relative ascribed interpretation (see Massaro et al., 2012).**

| **Measure** | **Description*** | **Interpretation** |
| --- | --- | --- |
| Time to first fixation | Time in seconds from when the stimulus was shown until the start of the first fixation within the cluster. | Used within the first formed cluster, it indexes the attraction power/saliency of the content of that particular cluster. The more framed the image (expected content), the longer the time to first fixation. |
| Fixation number | The number of the fixations within a cluster. | Richness of details. |
| Fixation duration | The length of the fixation duration in seconds within a cluster. | Salience/relevance of the content. |
| Observation number and duration | Number and duration of visits to a cluster. | Capacity of a cluster to capture attention; Salience/relevance of the content with respect to the other clusters // to the task. |

* As reported in the Tobii Studio 1.X – User Manual v. 1.0 [58, pp. 82-86]
