# Supplementary material for: Exploring Responses to Art in Adolescence: A Behavioral and Eye-Tracking Study
Source: PLoS One. 2014 Jul 21;9(7):e102888. doi: 10.1371/journal.pone.0102888 (PMC4105571; doi:10.1371/journal.pone.0102888)
Supplement: Table S2 — List of Paintings. (DOCX) [file pone.0102888.s002.docx]

**Table S2. List of Paintings.**

| **Category** | **Title** | **Artist** | **Year** | **Collection** |
| --- | --- | --- | --- | --- |
| **Human Dynamic** | The Flea | Crespi, Giuseppe Maria | 1707-09 | Galleria degli Uffizi, Florence |
|  | Richard Humphreys, the Boxer | Hoppner, John | XVIII sec. | The Metropolitan Museum of Art, New York |
|  | Apollo | Dossi, Dosso | 1524 | Galleria Borghese, Rome |
|  | Une jeune fille s'apprêtant à orner la statue de l'Amour d'une guirlande de fleurs | Roslin, Alexandre | 1783 | Musée du Louvre, Paris |
| **Human Static** | Portrait of an Artist in His Studio | Géricault, Théodore | c. 1820 | Musée du Louvre, Paris |
|  | The Countess of Chinchón | Goya Y Lucientes, Francisco De | 1800 | Museo del Prado, Madrid |
|  | Ferdinand IV, King of Naples | Mengs, Anton Raphael | 1760 | Museo del Prado, Madrid |
|  | Old Woman Seated | Puga, Antonio | XVII sec. | Museo del Prado, Madrid |
| **Nature Dynamic** | Le torrent (Tivoli) | Michallon, Achille-Etna | 1818-21 | Musée du Louvre, Paris |
|  | Cloud Study with Horizon | Dahl, Johan Christian Clausen | 1832 | Nationalgalerie, Berlin |
|  | Scogliera a Sestri Levante | Gignous, Eugenio | 1890 | Private Collection |
|  | The Waterfalls at Terni | Hackert, Jacob Philipp | 1779 | Private Collection |
| **Nature Static** | Fontainebleau: Oak Trees at Bas-Bréau | Corot, Camille | c. 1832 | The Metropolitan Museum of Art, New York |
|  | Bohemian Landscape with Mount Milleschauer | Friedrich, Caspar David | 1808 | Gemäldegalerie, Dresden |
|  | The Oaktree in the Snow | Friedrich, Caspar David | 1829 | Nationalgalerie, Berlin |
|  | Le Bas-Bréau, à Chailly | Leprince, Robert-Léopold | 1825 | Musée du Louvre, Paris |
